# Multiple Mechanisms Controlling Uterine Function: Editorial to Special Issue “Molecular Mechanisms of Uterine Receptivity for Embryo Implantation”

**DOI:** 10.3390/ijms242417629

**Published:** 2023-12-18

**Authors:** Jan Tesarik

**Affiliations:** MARGen Clinic, Camino de Ronda 2, 18006 Granada, Spain; jtesarik@clinicamargen.com

Uterine (endometrial) receptivity is the ability of the endometrium to successfully attach to the embryo, to promote its implantation, to nourish it and keep it alive. It describes the intricate process undertaken by the uterine lining to ensure embryo implantation, and its synchronization with embryo development is critical to the establishment of pregnancy [1]. While embryo development and endometrial preparation are concurrent yet independent processes, their synchronization is critical to the success of embryo apposition, adhesion, invasion, and further ongoing pregnancy [1].

Endometrial receptivity is controlled by the function of the corpus luteum (CL), the structure evolving from the ovarian follicle emptied during ovulation. The condition in which CL fails to support adequately uterine function is called CL deficiency (CLD). In most cases, CLD is related to the insufficient secretion of progesterone by CL, a condition which is relatively rare in natural ovulatory cycles, but is much more likely to occur in cycles, in which strong hormonal stimulation is used to enhance ovarian follicular growth, leading to hormonal imbalance. Luteoplacental shift, referring to the period during which CL function is progressively taken over by the placenta in the third month of gestation, is another critical event required for the continuation of pregnancy.

A decrease in the level of progesterone can be detected by repeated blood tests. However, when this condition is detected, it is sometimes too late for saving pregnancy. It is thus very important to elaborate sensitive and reliable molecular tools to detect this menace in advance. This Special Issue contains six papers in which molecular mechanisms of uterine receptivity and embryo implantation are examined from different points of view. Four articles describe original research and two are review ones.

The research article by Wu et al. focuses on the action of EGF to enhance the GnRH-II regulation of human decidual stromal cell motility and the involvement of Twist and N—cadherin signaling in this process. The results show that human decidual tissue and stromal cells express both EGF and GnRH-I receptors. Moreover, GnRH-II is shown to act through the GnRH-I receptor and to employ Twist and N-cadherin signaling to promote the motility of human decidual endometrial stromal cells. The authors conclude that molecules which regulate Twist and N-cadherin expression may become potential therapeutic targets in the treatment of infertility due to embryo implantation failure.

Data described in the research article by Yu et al. compare the microRNA expression profiles of exosomes derived from receptive (RL95-2) and non-receptive (AN3-CA) endometrial epithelial cell (EEC) lines to identify exosomal miRNAs closely linked to endometrial receptivity. According to these data, among the 466 differentially expressed miRNAs, miR-205-5p was the most highly expressed in exosomes secreted from receptive RL95-2 cells, and miR-205-5p, enriched at the adhesive junction, was closely related to endometrial receptivity. ZEB1, a transcriptional repressor of E-cadherin associated with endometrial receptivity, was identified as a direct target of miR-205-5p. Furthermore, administration of genetically modified exosomes overexpressing miR-205-5p mimicked upregulated E-cadherin expression by targeting ZEB1 and improved spheroid attachment of non-receptive AN3-CA cells, suggesting that the miR-205-5p/ZEB1/E-cadherin axis plays an important role in regulating endometrial receptivity. Accordingly, the use of exosomes harboring miR-205-5p can be considered a potential therapeutic approach for improving embryo implantation.

Scheliga et al. performed a study to evaluate molecular effects of endometrial scratching (ES), a technique sometimes used with the aim to improve uterine receptivity. They analyzed proteome changes evoked by this intervention in fertile women. The rationale of the recourse to ES is the hypothesis that it may trigger a local immune response, leading to an improved endometrial receptivity through modifications of the gene expression of cytokines, growth factors, and adhesive proteins, potentially modulating inflammatory pathways and adhesion molecule expression. The data suggest that ES probably has an impact on the proteins involved in immune response pathways and cytoskeleton formation, which could potentially increase endometrial receptivity. Specifically, proteins that are involved in the immune response and cytoskeleton regulation showed a trend toward higher abundance after the first ES. On the other hand, proteins with a decreasing abundance after the first ES play roles in the regulation of the actin cytoskeleton and cellular processes such as intracellular transport, apoptosis, and autophagy. Since these trends in protein changes suggest that ES may affect extracellular matrix remodeling potentially favorable to embryo implantation, the results of this pilot study can serve a basis for further research as to the real benefits of ES in women with previous implantation failures.

Original research data presented by Vanstokstraeten et al. present a comparison of vaginal and endometrial microflora in order to evaluate to what extent vaginal and cervical microbiomes can cross-contaminate endometrial samples, resulting in a biased representation of the endometrial microbiome as a cause of decreased uterine receptivity. In fact, in a clinical setting, endometrial samples are always collected by passing through the vaginal–cervical route. By using culturomics to compare endometrial biopsy samples, obtained by diagnostic hysteroscopy, with vaginal swabs from the same subfertile women, the authors conclude that, even though cross-contamination derived from sampling cannot be completely excluded, the data suggest the potential existence of a unique endometrial microbiome that is not merely a presentation of cross-contamination. In practical terms, this means that, even with some reservation, culturomics analysis of endometrial biopsy from infertile and subfertile women should be seriously taken into account while choosing the optimal treatment strategy.

The paper by Lazim et al. provides a systematic review on the expression of *HOXA10* gene (a member of homeobox gene family) in women with endometriosis. Though expected not to be related directly with the uterine receptivity, endometriosis is a serious condition causing female infertility. The paper by Lazim et al. shows the *HOXA10* gene to be downregulated in most studies performed in women with endometriosis as compared with women without endometriosis. The authors suggest that, in addition to other negative effects on oocyte quality and quantity, the endometriosis-related downregulation of the *HOXA10* gene may be related to the impaired development of pinopodes in the endometrium during the luteal phase, thus affecting directly the uterine receptivity.

The systematic review and meta-analysis by Huang et al. resumes the role of non-coding RNAs as biomarkers for embryo quality and pregnancy outcomes. Embryo quality is an important factor that must be seriously taken into account, in addition to the uterine receptivity, to predict in vitro fertilization (IVF) outcome. Thus, this paper is also involved in this Special Issue. Despite advances in in vitro fertilization (IVF), there is still a scarcity of non-invasive and reliable biomarkers for selecting embryos with the highest developmental and implantation potential. Small non-coding RNAs (sncRNAs) are excreted into biological fluids and culture media by oocytes and preimplantation embryos and can thus be studied without causing any kind of damage to the cells. The authors analyzed 18 studies that met their selection criteria and identified 22 and 47 different sncRNAs to be dysregulated in follicular fluid (FF) and spent culture media (SCM), respectively. The association with IVF failure was most constantly detected for four of the sncRNAs. In fact, *MiR-663b*, *miR-454* and *miR-320a* in FF and *miR-20a* in SCM showed consistent dysregulation in two different studies. Further study is recommended as to the use of these sncRNAs as noninvasive markers of oocyte and embryo quality.

In conclusion, the articles published in this Special Issue do not provide immediate solutions as to the diagnosis and treatment of female infertility, but they are of enormous value in view of orienting further research whose results may be available in the near future.
